# Art skill-based rehabilitation training for upper limb sensorimotor recovery post-stroke: A feasibility study

**DOI:** 10.1177/02692155221105586

**Published:** 2022-05-31

**Authors:** April Christiansen, Marta Scythes, Benjamin R Ritsma, Stephen H Scott, Vincent DePaul

**Affiliations:** 1 Centre for Neuroscience Studies, Queen’s University, Kingston, ON, Canada; 2 Haliburton School of Art and Design, 125171 Fleming College, Haliburton, ON, Canada; 3 Department of Physical Medicine and Rehabilitation, 4257 Queen’s University, Kingston, ON, Canada; 4 Department of Biomedical and Molecular Sciences, 4257 Queen’s University, Kingston, ON, Canada; 5 Department of Medicine, Queen’s University, Kingston, ON, Canada; 6 School of Rehabilitation Therapy, Queen’s University, Kingston, ON, Canada

**Keywords:** Stroke, rehabilitation interventions, upper extremity (arm), functional restoration program

## Abstract

**Objective:**

The objective of this study was to assess the feasibility of delivering Art skill-based Rehabilitation Training (ART), a novel upper limb motor training program, to patients with stroke as an adjunct to standard care in an inpatient setting.

**Design:**

Feasibility study.

**Setting:**

Inpatient stroke rehabilitation unit at a university hospital.

**Participants:**

Thirty-eight patients admitted to a stroke rehabilitation unit with upper limb motor impairment were enrolled in the ART program facilitated by trained non-healthcare professionals between December 2017 and June 2021.

**Intervention:**

The ART program included nine, one-hour sessions of supervised tracing and freehand drawing tasks completed with both hands. This program was intended to be delivered at a frequency of three times per week over a duration of 3 weeks or for the length of inpatient stay.

**Main outcome measures:**

Feasibility outcomes included ART program adherence, acceptability, and safety.

**Results:**

Thirty-two (84%) participants with subacute stroke completed the ART program and 30 (79%) were included in the study analysis. Participants completed 93–100% of the ART tasks in a median [IQR] of 8 [6–10] ART sessions over a median [IQR] duration of 15 [7–19] days. ART program facilitators effectively provided upper limb assistance to patients with more severe upper limb impairments. Adherence and acceptability were high and no study-related adverse events occurred.

**Conclusion:**

The ART program was feasible to deliver and highly acceptable to patients with stroke. Further research is warranted to explore the impact of ART on upper limb sensorimotor function and use.

## Introduction

Over the past 25 years, the development of new stroke rehabilitation interventions has largely been driven by the promise of experience-dependent neuroplasticity. In constraint-induced movement therapy (CIMT), intensive task-based training with the more affected limb is combined with constraining the less affected limb to promote adaptive (relearning functional and efficient movement of the affected limb) rather than maladaptive neuroplastic changes (e.g. learned non-use, compensation). Similarly, robotic interventions are designed to allow patients to achieve higher repetitions of goal-oriented movements during rehabilitation. In other research, supplementing standard rehabilitation care with additional upper limb-focused, therapist-delivered sessions have been shown to increase upper limb training intensity, with patients achieving up to 289 purposeful movement repetitions within a single 60-minute session.^1,2^ Although many of these novel interventions have promising effect sizes at both the activity and impairment levels,^3^ broad translation to clinical practice has been suboptimal. Dependence on expensive technology, or the availability of highly skilled physiotherapists and occupational therapists, can limit the practical application of these intervention approaches. As such, patients in inpatient stroke rehabilitation settings continue to spend many hours per day physically inactive, alone or involved in non-therapeutic activities.^4^ There is a need for approaches that optimize neural and functional recovery through high repetitions of meaningful, arm and hand-focused activities, that can be delivered by non-professionals, and that motivate patients to continue to practice outside of supervised sessions and beyond their stay in rehabilitation.

Emerging neuroscience and rehabilitation research suggests the potential of visual art modalities in stroke rehabilitation.^5^ Artistic drawing is one of the most complex human behaviors requiring concurrent involvement of several cognitive and sensorimotor domains. Intensive artistic drawing has been shown to result in functional and structural changes in brain regions associated with visuospatial processing, planning, sequencing, and execution of complex motor tasks.^6,7^ In the context of stroke rehabilitation, artistic drawing is consistent with the neurorehabilitation and motor learning principles of task-oriented practice, challenge, salience, task-repetition, engagement, and action observation which have been evidenced to optimize the acquisition, retention, and generalization of motor skills.^8^ Despite the indication of artistic drawing practice in stroke rehabilitation, there are currently no clear design considerations for art-based upper limb training approaches that utilize drawing as a primary modality.

The purpose of this study was to assess the feasibility of delivering Art skill-based Rehabilitation Training (ART), an original, supplemental upper limb training program, to patients with stroke in an inpatient rehabilitation setting. ART was designed to augment upper limb training intensity through engaging patients in directed, repetitive, graded drawing tasks.

## Methods

A convenience sample of eligible participants was invited to participate in the Art skill-based Rehabilitation Training (ART) program. Participants were recruited from the inpatient stroke rehabilitation unit at Providence Care Hospital (PCH), a tertiary academic hospital affiliated with Queen's University in Kingston, Canada by an on-site physiotherapist not involved in their care. Inclusion criteria were as follows: adult (≥ 18 years of age), experienced an ischemic or a hemorrhagic stroke 2 weeks to 6 months prior, able to comprehend and follow instructions, had a clinician-identified upper limb motor impairment in the healthcare record, no other neurological conditions, and able to sit in a chair with a backrest for approximately 1 hour. The study was reviewed and approved by Queen's University Research Ethics Board and Providence Care Hospital. All participants provided informed consent.

The feasibility of delivering ART as an adjunct to standard care rehabilitation was determined by examining program adherence, acceptability, and safety. High levels of adherence, acceptability, and safety were considered indicative of the ART program's success. Adherence to the ART program was determined by the following: (1) completion of the prescribed ART tasks, (2) number of sessions participants required to complete the ART program, and (3) number of days participants were enrolled in the ART program (duration of ART). Acceptability was assessed using a 12-question, 5-point Likert-type scale questionnaire completed by participants at the end of the ART program to assess program satisfaction, intent to continue use, and perceived appropriateness. The safety of ART was determined by the number of adverse events that were reported during or after the ART program by participants. An adverse event was defined as any new onset symptom or condition such as pain, fatigue, muscle soreness, or injury, occurring during or following ART participation.

Clinical assessments were conducted to characterize the study participants. Clinical assessments included the Functional Independence Measure (FIM) to measure participant's functional abilities^9^; the Montreal Cognitive Assessment (MoCA) to screen for cognitive impairment^10^; the Behavioral Inattention Test (BIT) to assess the presence of visuospatial neglect^11^; the Purdue Pegboard Test (PPT) to measure manual dexterity and gross hand/arm movement^12^; as well as the Chedoke-McMaster Stroke Assessment (CMSA)^13^ and Fugl-Meyer Assessment for Upper Extremity (FMA-UE) to assess impairments of the arm and hand.^14^ Handedness of participants was assessed using the modified Edinburgh Handedness Inventory.^15^ All clinical assessments were performed by a physiotherapist within one week before and after ART enrollment or completion. Participants were categorized based on the stroke-affected side of their body; the most impaired upper limb was labeled “affected” and the other upper limb was denoted “unaffected.”

The ART program was designed to enhance functional recovery of the arm and hand through experience-dependent neuroplasticity, considering the following principles: (1) *Intensity of training.* Intensity may be operationalized as the number of repetitions performed within the allotted training time. Intense training may facilitate the restoration of function through repetitively engaging relevant neural substrates and driving plastic changes in the primary motor cortex.^16^ For patients with stroke, the provision of additional, high-volume upper limb training has shown to effectively increase training intensity and upper limb recovery.^17,18^ ART aims to increase training intensity through the provision of extra upper limb practice for one hour, three days per week as this has shown to be an acceptable schedule for stroke survivors.^19,20^ (2) *Task-oriented practice*. Task-oriented training is a form of motor learning that focuses on skill acquisition in the context of a particular functional activity. Skill is the ability to achieve a goal with consistency, flexibility, and efficiency. Functional magnetic resonance imaging (fMRI) studies have demonstrated that repetitive, task-oriented practice with the impaired limb stimulates neuroplastic changes that remediate contralateral maladaptive brain patterns and facilitate motor learning, as indicated by the laterality index, whereas these patterns appear to be absent in repetitive but non-task-oriented training.^21,22^ Following subacute stroke, task-oriented training has been demonstrated to confer greater arm and hand functional gains compared to conventional, non-task-oriented exercise.^23^ ART focuses on enhancing drawing skills with the goal of consistently completing the given ART tasks. The fine and gross motor skills developed with artistic drawing may be transferable to other functional activities in similar contexts.^24^ (3) *Challenge.* Challenge refers to task difficulty with respect to the skill of the performer. Repetitive practice of a task or movement with continual success has no advantage for learning and may allow for compensatory movements to develop in the case of stroke, which interferes with long-term upper limb recovery.^25^ Repetitive practice of challenging movements is thought to optimize sensorimotor feedback and prediction, allowing for effective motor skill acquisition.^26^ The ART tasks and modules become progressively more difficult, requiring more complex motor techniques. (4) *Engagement.* Engagement is the deliberate effort and commitment to working toward personal goals and active participation in rehabilitation, which may be maximized by providing patients with enjoyable, meaningful tasks.^27^ Previous research has emphasized that there is a clear relationship between enjoyment, engagement, and functional improvement post-stroke.^28^ Creating visual art has demonstrated to be an enjoyable process for many stroke survivors,^5^ suggesting that artistic training modalities may enable greater patient engagement in rehabilitation activities. ART aims to facilitate patient engagement in additional upper limb task practice through the provision of appealing and recognizable drawing tasks. (5) *Action Observation.* Observational motor learning, also coined “modeling,” posits that an unskilled learner benefits from observing a more skilled performer demonstrate the desired motor action as the observer can attend to and code the movement pattern and the goal of the motor task.^29^ Observing an action be performed by another person has been shown to increase activation in motor-related brain areas in stroke survivors.^30^ A recent randomized controlled pilot trial found that patients with subacute stroke who observed video demonstrations of a task prior to performance had greater post-treatment clinical scores compared to patients who only performed the task with no video demonstration.^31^ ART draws upon the concept of action observation by encouraging participants to observe the motor actions of a skilled artist via a video demonstration prior to completing each task.

**Figure 1.**
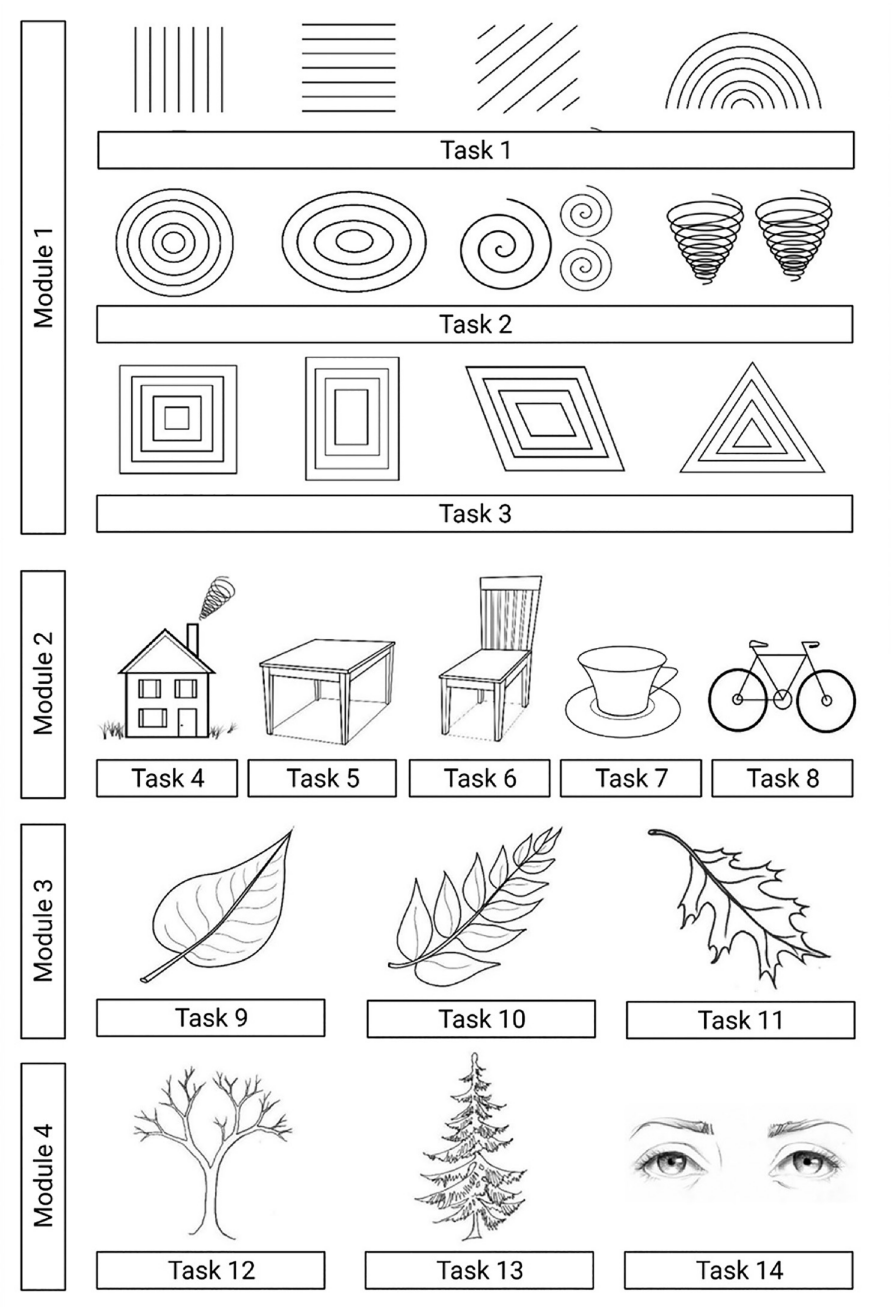
Art skill-based rehabilitation training (ART) tasks in each module.

**Figure 2.**
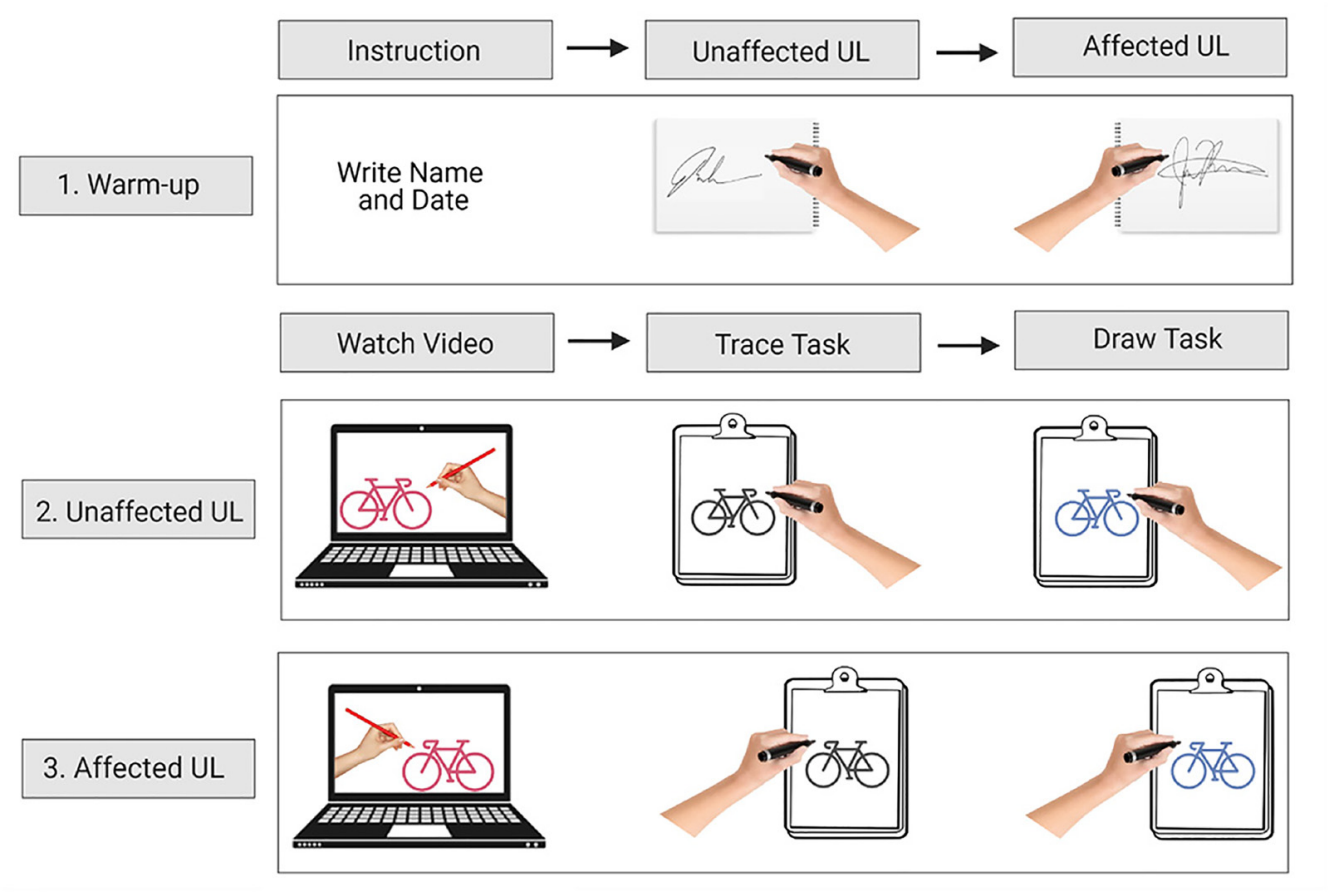
Art skill-based rehabilitation training (ART) task completion process.

The ART program consists of four modules, with a total of 14 tasks, that progress in difficulty (Figure 1). Participants began each session by writing their name and date with both hands in their personalized notebook using a thin pencil or large pen, and a grip tool if necessary (Figure 2). Prior to the physical performance of the drawing, participants watched an instructional video of the ART task with the facilitator on a laptop computer. The video demonstrated an artist freehand drawing the task with their right or left hand, selected to match the affected or unaffected hand of the participant. The participant then completed a tracing and freehand drawing of the task on a separate blank, white piece of 8.5″ by 11″ paper.

Participants were instructed to trace (copy) each ART task prior to attempting to freehand draw, as learning how to accurately copy the shape and spatial position of a visual element is the first step in learning how to freehand draw.^32^ The task tracings also served as an opportunity to increase the number of motor repetitions provided by a given task. After completion of the task tracing and freehand drawing with their unaffected arm, participants repeated the video instruction, task tracing, and drawing with their affected arm.

Both upper limbs were trained to promote interlimb transfer. The practice of a novel motor task with one arm often leads to an improvement in subsequent performance with the other arm.^33^ For patients with upper limb impairment following stroke, task-oriented practice with both upper limbs has shown to enhance interlimb transfer and positively influence functional recovery.^34^

Participants completed all drawing tasks while sitting at a dining-style table with a smooth surface. Both arms were placed on the table with the drawing paper in front. Both upper limbs were engaged in the task, one to draw and one to either stabilize the clipboard with the paper or to provide overall stability on the table. Participants could position the clipboard/paper as they wished to best complete the task. If participants were unable to hold the clipboard in place, the ART facilitator was able to do so. If participants were unable to complete the ART tasks independently, they were provided with manual support of the arm at the elbow and forearm, in addition to hand-over-hand assistance to grip the writing tool, following an “assistance as needed” approach,^35^ providing as much support as necessary and as little as possible to maximize participant engagement without compromising motivation or effort. In the execution of an assisted ART task, the participant was instructed to first attempt to complete the task as much as able. Sitting on the patient's weaker side, after giving the patient time to initiate and begin the movements, the facilitator provided just enough assistance to complete the specific task.

The ART program was designed to have nine one-hour sessions delivered at a minimum of three days per week, over a three-week duration by a trained ART facilitator. This training schedule has shown to be a pragmatic approach to the delivery of additional, upper limb training and result in a high level of participant adherence.^19,20^ It was anticipated that a more demanding protocol would be a barrier to participant recruitment, engagement, and retention.

ART was scheduled between 8 a.m. and 5 p.m. on weekends or weekdays with consideration given to the participants’ preferences for training, timing of family visits, standard therapies, and other daily activities (e.g. bathing, dressing, eating). All participants also completed occupational therapy and physiotherapy five days per week and speech-language therapy when applicable.

Two trained facilitators (non-healthcare professionals) delivered ART one-on-one to participants. The primary facilitator (MS) led the development and implementation of the ART program. Prior to independent delivery of ART to participants, MS received instruction and supervised experiential practice related to working with individuals with stroke, including handling the hemiplegic limb, from an experienced occupational therapist. The second facilitator (AC), a graduate student in clinical neuroscience, was trained to deliver the ART program by the primary facilitator. ART facilitator training involved 8 hours of didactic and experiential learning related to the safe delivery of ART.

All aspects of the ART program design (i.e. tasks, modules, videos, delivery schedule) were developed by a professional fine artist and medical illustrator (MS) in consultation with stroke survivors, clinicians (i.e. occupational therapists, physiotherapists, physiatrists), and researchers with expertise in stroke rehabilitation, neuroscience, and motor learning.

Descriptive statistics (median, interquartile range [IQR]) were used to calculate ART task completion rates and describe clinical variables. Frequency statistics were used to summarize questionnaire data. All analyses were completed using IBM SPSS Statistics for Windows, version 26.

## Results

A total of 38 participants enrolled in the ART program and 32 completed the intervention, yielding an 84% program retention rate. Two participants withdrew from the study. One participant withdrew prior to completing 50% of the program due to general fatigue. The other withdrew due to disinterest in the ART program after completing the first task. Four participants were discharged from the hospital prior to completing 50% of the ART program. Thirty participants were included in the study analysis as two participants who completed the ART program were excluded due to missing baseline clinical and demographic data.

Detailed participant demographics and baseline clinical data can be found in Table 1. The majority of participants were male (76.7%) and right-hand dominant (86.7%). Two participants scored as ambidextrous on the Edinburgh Handedness Inventory, but each reported being left and right hand dominant, respectively.

**Table 1.** Participant demographics and baseline clinical data.

Characteristic/measure	ART (*N* = 30)
Age (y)^a^	69 [63–76]
Sex (F/M)	7/23
Handedness (R/L/A)	26/2/2
Affected UL (R/L)	13/17
Visuospatial deficits (Y/N)	9/21
Aphasia (Y/N)	3/27
Stroke type (I/H)^b^	23/6
Stroke location (C/S/C + S/Cr/B/S + Cr)^b^	8/7/6/2/4/1
Days post-stroke^a,c^	32 [26–42]
Days in ART program^a^	15 [7–19]
Number of ART sessions^a^	8 [6–10]
FIM^a^	90 [63–76]
MoCA^a^	22 [21–26]
BIT^a,b^	141 [135–144]
CMSA affected UL^a,b,d^	8 [4–10]
CMSA unaffected UL^a,b,d^	13 [12–13]
PPT affected UL^a^	2 [0–6]
PPT unaffected UL^a^	9 [8–11.5]

y: years; F/M: female/male; R/L/A: right/left/ambidextrous; UL: upper limb; Y/N: yes/no; I/H: ischemic/hemorrhagic; C: cortical; S: subcortical; B: brainstem; Cr: cerebellar; FIM: Functional Independence Measure; MoCA: Montreal Cognitive Assessment; BIT: Behavioral Inattention Test; NIHSS: National Institutes of Health Stroke Scale. PPT: Purdue Pegboard Test; CMSA: Chedoke McMaster Stroke Assessment. CMSA arm + hand scores from the impairment inventory, quantified using a 7-point scale based on Brunnstrom’s stages of recovery.

^a^ Median value [interquartile range].

^b^ *N* = 29.

^c^ Days post-stroke at time of first ART session.

^d^ Twenty-three CMSA scores were converted from FMA-UE scores.

## Feasibility

The 30 participants were a median [IQR] of 32 [26–42] days post-stroke when beginning the ART program, were enrolled in the program for a median [IQR] of 15 [7–19] days, and required a median [IQR] of 8 [6–10] sessions to complete the program (Table 1).

**Figure 3.**
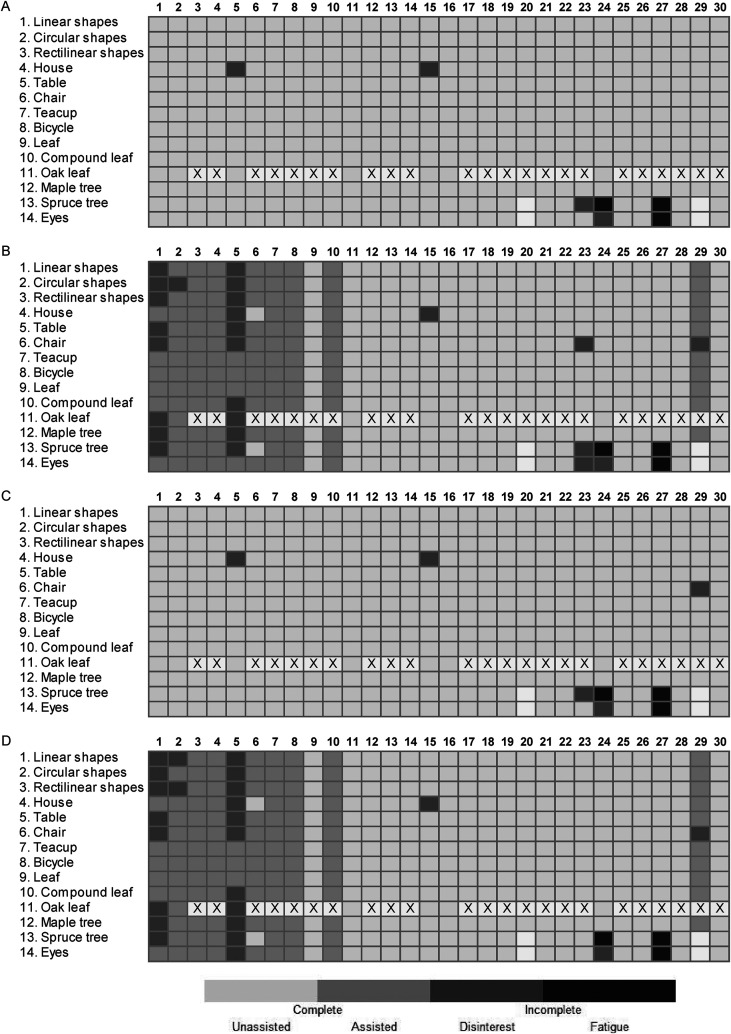
Art skill-based rehabilitation training (ART) task completion. (A) Completion of task tracings with unaffected upper limb. (B) Completion of task tracings with affected upper limb. (C) Completion of task drawings with unaffected upper limb. (D) Completion of task drawings with affected upper limb. Tasks are indicated on the vertical axis in the order of completion. Columns denote participant performance of the 30 participants. Light cells indicate the task was completed without assistance. Light gray cells indicate that the task was completed with manual assistance. Gray cells indicate that the participant did not complete the task due to disinterest. Dark gray cells indicate that the participant did not complete the task due to fatigue. A number of participants who completed each task are listed on the right vertical axis, including tasks completed with or without assistance. X marks tasks that were not delivered to the participant.

For the unaffected upper limb, the median [IQR] completion rates for all task tracings and freehand drawing tasks were 100% [98–100%] and 100% [96–100%], respectively (Figure 3A and B). The median [IQR] completion rates for all tasks completed with the affected upper limb were 93% [84–98%] for the tracings and 93% [89–98%] for the freehand drawings (Figure 3C and D). Two participants were discharged from the hospital early, after completing over 50% of the program, leading to incompletion of the last two tasks. Seven participants did not complete all task tracings or drawings due to disinterest in a task or fatigue. Ten participants required physical assistance to complete tasks with their affected upper limbs. The oak leaf (task 11) was omitted after seven participants had completed the program due to verbal feedback from these participants that the oak leaf was too challenging. Evidence suggests that when task difficulty surpasses one's level of developed skill or perceived ability to succeed, there can be detrimental effects on performance, rehabilitation engagement, self-efficacy, and confidence.^36^ The ART aims to foster confidence and engagement; thus, the research team felt that this was a necessary program revision. The oak leaf was not included in the calculation for the task completion rates for the other 23 participants (Figure 3).

Twenty-eight (93%) participants completed the end-of-study questionnaire. ART was highly acceptable to participants, with 26 (93%) participants rating their level of satisfaction a 4 or 5 on a 5-point scale, and two participants concluding that they were “somewhat satisfied” (three out of five on the Likert scale). All participants (100%) reported that they would recommend the program to other stroke survivors devoid of program modifications (Supplemental Material). Twenty-one (75%) participants indicated that the ART program “very much” motivated them to use their affected upper limb, and 71% specified that the ART session duration of one hour was “just right.” Over 60% of participants found the task progression, tracing exercises, and video demonstrations to be useful. A majority of participants (> 86%) found ART to be tolerable with minimal pain; no participants rated ART as “painful.”

Five participants with documented hemiplegic shoulder pain and limited range of motion prior to starting the ART program indicated that they experienced some pain during or after the ART sessions in their affected shoulder (Supplemental Material); however, pain did not lead to discontinuation of an ART task or session by any participant. Fatigue was reported by two participants leading to task incompletion (Figure 3). Post-stroke fatigue was noted in these two participants by a physiatrist outside of ART sessions. There were no reports of injuries, muscle soreness, or serious adverse events.

Scheduling conflicts occurred on six separate occasions for five participants when participants had unplanned changes to standard care therapy schedules. Thus, while the program was designed with a set number of tasks to be completed over a three-week duration at a frequency of three times per week, periodic modifications were made to accommodate some participant-specific needs and circumstances.

## Discussion

Our findings from this study suggest that it is feasible to deliver ART to patients with upper limb motor impairment in an inpatient stroke rehabilitation setting. Participants with an array of stroke-related impairments were able to successfully complete repetitive, challenging drawing tasks with both upper limbs with or without manual assistance provided by a facilitator. Participants completed several ART sessions per week at their desired pace over a three-week period or until hospital discharge, reported high levels of program satisfaction, and no adverse events occurred beyond shoulder pain and fatigue that were present prior to initiating ART.

A critical finding from our study was that participants with more severe upper limb functional deficits, as evidenced by the low baseline combined arm and hand CMSA scores of two or three out of fourteen, were capable of participating in ART. This highlights that ART is an inclusive rehabilitation option as even those with little to no voluntary upper limb movement are able to partake in the program.^13^ Those with mild to moderate upper limb deficits frequently have a variety of rehabilitation options available to them, such as traditional therapy exercises and recreational activities, while the activity options for those with more severe deficits are limited. For example, constraint-induced movement therapy (CIMT) requires participants to have active wrist and finger movement, allowing for only 10% of inpatients with stroke to be eligible for this treatment.^37^ The ART protocol appears to be feasible for a diverse clinical population of individuals with minimal distal movement to participate in, as the facilitator provides upper limb support and assistance while the patient attempts to perform the task. It should be addressed that voluntary motor control of the wrist and fingers, particularly extension, is a key factor determining the potential for clinically meaningful change, as motor improvements with intensive upper limb training are mainly based on adaptations through learning to optimize the use of intact end-effectors.^37^ Residual motor capability, although an important factor, is certainly not the only factor that influences the use of the impaired hand post-stroke. Participation in rehabilitation can foster confidence and self-efficacy which can encourage the use of the impaired upper limb^38^; thus, there is still benefit for patients with minimal volitional control.

Interventions which include participants with more severe upper limb deficits frequently require additional support or supervision from a therapist, which is a key barrier in the implementation of supplementary stroke rehabilitation programs.^39^ As healthcare professionals face increasing demands of their time, exacerbated by system-based fiscal constraints and patient complexity, trained non-healthcare professionals, or by extension volunteers, are an essential resource to enhance patient support in stroke rehabilitation.^40^ In the current study trained, non-healthcare professionals (ART facilitators) were able to safely and effectively deliver a rehabilitation intervention to patients with upper limb impairments and other complicated, co-existing deficits using manually guided assistance when necessary. This is a unique feature of the ART program and this study as there is limited literature evaluating the delivery of additional exercise programs by trained, non-healthcare professionals in stroke rehabilitation. It is worth acknowledging that the effective incorporation of non-healthcare providers or volunteers to extend the reach of clinicians in clinical contexts requires careful consideration due to issues related to patient safety, confidentiality, reliability, role overlap, limited resources, and communication between these individuals and the clinical team.^40^ In light of this, future research examining the role of volunteers in the delivery of stroke rehabilitation training, such as ART, is warranted.

Cognitive impairments are a common sequelae of stroke and can greatly affect functional recovery through reduced participation in rehabilitation. Numerous studies have identified that even mild impairments in cognitive faculties (e.g. MoCA < 26) can lead to significantly lower levels of participation in stroke rehabilitation across all activity domains (e.g. low-demand tasks, high-demand tasks, social/educational).^41^ Patient characteristics, provider perceptions, practice guidelines, and available treatment options are all variables that may impact the relationship between cognitive impairment and rehabilitation participation. For instance, some rehabilitation programs may not be designed to effectively respond to cognitive impairment, or stroke-affected individuals with cognitive deficits may not be provided the same level of rehabilitation if they are perceived as poor candidates.^42^ The ART program was mindfully designed in consideration of individuals with varying neurocognitive post-stroke deficits and we were able to successfully include and engage survivors with mild (e.g. MoCa < 26) and moderate (e.g. MoCA 11–17)^10^ cognitive impairment in directed drawing tasks.

Completion rates of the ART tasks were high for both the affected and unaffected upper limbs. Reasons for task incompletion or study withdrawal were consistent with previously published stroke rehabilitation investigations − fatigue, scheduling conflicts, or lack of interest.^43^ In this study, no tasks were requested to be stopped by participants due to pain. Two participants requested to stop a task or session due to fatigue, and six requested to skip a tracing or drawing task out of disinterest. General fatigue and disinterest in the ART program led to withdraw of two participants. Importantly, post-stroke fatigue and disinterest in exercise training have been previously identified as impediments to rehabilitation program delivery and participation.^44^ As well, fatigue during training may not be considered a true adverse event and may rather provide evidence of achieving a practice intensity target or limit of tolerance.

When interpreting the results, some limitations require consideration. The feasibility of ART was evaluated in a small convenience sample from a single inpatient facility which may limit the generalizability of the results. The ART program was designed with a set number of tasks to be completed over a specific frequency and duration; however, this training schedule was not precisely adhered to due to scheduling conflicts or early hospital discharge, meaning there are some limitations in determining the feasibility of the intended delivery structure. The research team felt that this was a necessary trade-off, which gave participants the flexibility to participate in ART regardless of the length of their inpatient stay or therapy schedule. The number of patients eligible and recruited to participate in ART were not recorded in this study, but these parameters are relevant to the feasibility of implementing an intervention in a rehabilitation setting; therefore, these parameters should be formally documented in future evaluations of ART. As ART was designed to intensify upper limb rehabilitation, it is also recommended that future trials conduct a detailed analysis of the duration of upper limb use and a number of active motor repetitions generated during a timed ART session, quantified by direct observation and/or upper limb-mounted accelerometers.

This study demonstrated the feasibility of delivering ART as an adjunct to standard inpatient stroke rehabilitation with non-healthcare professional facilitation. Participants with complex and varied upper limb post-stroke impairments were able to complete the ART program with no significant adverse events and reported high levels of program satisfaction. We postulate that the use of ART as an adjunct to existing standard care therapy may be one way to effectively augment upper limb activity, recovery, and patient engagement in rehabilitation; however, future investigation is warranted.

**Clinical messages**

- An artistic training approach, involving directed drawing tasks, appears to be feasible and safe for use in inpatient stroke rehabilitation.
- ART, as an adjunct to standard care therapy, may help augment upper limb training intensity by actively engaging patients in challenging, repetitive, and enjoyable drawing tasks.

## Supplemental Material

sj-docx-1-cre-10.1177_02692155221105586 - Supplemental material for Art skill-based rehabilitation training for upper limb sensorimotor recovery post-stroke: A feasibility study Click here for additional data file. Supplemental material, sj-docx-1-cre-10.1177_02692155221105586 for Art skill-based rehabilitation training for upper limb sensorimotor recovery post-stroke: A feasibility study by April Christiansen, Marta Scythes, Benjamin R Ritsma, Stephen H Scott and Vincent DePaul in Clinical Rehabilitation
