# Supplementary material for: Art skill-based rehabilitation training for upper limb sensorimotor recovery post-stroke: A feasibility study
Source: Clin Rehabil. 2022 May 31;36(11):1476–88. doi: 10.1177/02692155221105586 (PMC9515518; doi:10.1177/02692155221105586)
Supplement: sj-docx-1-cre-10.1177_02692155221105586 - Supplemental material for Art skill-based rehabilitation training for upper limb sensorimotor recovery post-stroke: A feasibility study [file sj-docx-1-cre-10.1177_02692155221105586.docx]

**Supplemental Material**

**Participant end-of-study questionnaire data**

**Participant satisfaction rating.**

| Question | n | 1   Not Satisfied | 2 | 3  Somewhat Satisfied | 4 | 5   Greatly Satisfied |
| --- | --- | --- | --- | --- | --- | --- |
| How satisfied are you with the program? | 28 | 0 | 0 | 2 | 4 | 22 |
| Question | n | 1  No | 2 | 3  Yes, with changes | 4 | 5  Yes |
| I would recommend this program to other stroke survivors. | 28 | 0 | 0 | 0 | 6 | 22 |

**Participant pain/discomfort rating.**

| Question | n | 1  Painful | 2 | 3  Some pain/discomfort | 4 | 5  Not painful |
| --- | --- | --- | --- | --- | --- | --- |
| I felt pain/discomfort during the sessions. | 28 | 0 | 0 | 2 | 2 | 24 |
| I felt pain/discomfort after the session. | 28 | 0 | 1 | 1 | 0 | 26 |

**Participant ratings of training intensity.**

| Question | n | 1  Too short/ long | 2 | 3 | 4 | 5  Just right |
| --- | --- | --- | --- | --- | --- | --- |
| The duration of each session was: | 28 | 0 | 1 | 3 | 4 | 20 |

**Perceived utility of ART**.

| Question | n | 1  Not helpful | 2 | 3  Somewhat helpful | 4 | 5  Very helpful |
| --- | --- | --- | --- | --- | --- | --- |
| The videos (watching an artist draw) were helpful. | 28 | 0 | 0 | 4 | 5 | 19 |
| The tracing exercises were helpful. | 28 | 1 | 0 | 0 | 10 | 17 |
| The progression from simple to complex objects was helpful. | 28 | 0 | 0 | 3 | 4 | 21 |

**Participant motivation ratings.**

| Question | n | 1  Not at all | 2 | 3  Sometimes | 4 | 5  Very much |
| --- | --- | --- | --- | --- | --- | --- |
| The program motivated me to use both of my hands/arms. | 28 | 0 | 0 | 5 | 6 | 17 |
| The program motivated me to use my weaker hand/arm. | 28 | 0 | 1 | 3 | 3 | 21 |
| Question | n | 1  Not at all | 2 | 3  Sometimes | 4 | 5  Very often |
| I practiced/did extra artwork between sessions. | 28 | 7 | 4 | 7 | 3 | 7 |

**Concluding remarks.**

| Question | n | 1  No | 2 | 3  Possibly | 4 | 5  Yes |
| --- | --- | --- | --- | --- | --- | --- |
| Do you think you may continue to do art once discharged? | 28 | 2 | 1 | 16 | 3 | 6 |
